# Can cartilage intermediate layer protein 1 (CILP1) use as a novel biomarker for canine myxomatous mitral valve degeneration levels or not?

**DOI:** 10.1186/s12917-023-03583-7

**Published:** 2023-03-07

**Authors:** Hyeon-Jin Kim, Jihyun Kim, Soomin Kim, Ha-Jung Kim

**Affiliations:** 1 Department of Internal Medicine, College of Veterinary Medicine, Chonnam National University, 77 Yongbong-Ro, Buk-Gu, Gwangju, 61186 South Korea; 2 BK 21 Project Team, College of Veterinary Medicine, Chonnam National University, Gwangju, 61186 South Korea

**Keywords:** Myxomatous mitral valve degeneration, Cartilage intermediate layer protein 1, Biomarker, Cardiac, Dogs

## Abstract

**Background:**

Myxomatous mitral valve degeneration (MMVD) is the most common degenerative heart disease in dogs and is associated with irreversible changes in the valve tissue. Although traditional cardiac biomarkers are efficient for diagnosing MMVD, there are limitations, therefore, it is important to find novel biomarkers. Cartilage intermediate layer protein 1 (CILP1), an extracellular matrix-derived protein, acts as a transforming growth factor-β antagonist and is involved in myocardial fibrosis. This study aimed to evaluate serum CILP1 levels in canines with MMVD. Dogs with MMVD were staged according to the American College of Veterinary Internal Medicine consensus guidelines. Data analysis was performed using the Mann–Whitney U test, Spearman’s correlation, and receiver operating characteristic (ROC) curves.

**Results:**

CILP1 levels were elevated in dogs with MMVD (*n* = 27) compared to healthy controls (*n* = 8). Furthermore, results showed that CILP1 levels were significantly higher in stage C group dogs compared to healthy controls. The ROC curve of CILP1 and NT-proBNP were good predictors of MMVD, although no similarity was observed between the two. Left ventricular end-diastolic diameter normalized to the body weight (LVIDdn) and left atrial to aorta dimension (LA/Ao) showed a strong association with CILP1 levels; however, no correlation was observed between CILP1 levels and vertebral heart size (VHS) and vertebral left atrial score (VLAS). The optimal cut-off value was selected from the ROC curve and dogs were classified according to the cut-off value (1.068 ng/mL, sensitivity 51.9%, specificity 100%). Results showed a significant association of CILP1 with cardiac remodeling indicators, such as VHS, VLAS, LA/Ao, and LVIDdn.

**Conclusions:**

CILP1 can be an indicator of cardiac remodeling in canines with MMVD and therefore, can be used as an MMVD biomarker.

## Background

Myxomatous mitral valve degeneration (MMVD) is the most common cardiac disease in dogs, accounting for 75–80% of total cases [1]. The prevalence of MMVD is high in small to medium-sized dog breeds (< 20 kg), including Cavalier King Charles Spaniel, Dachshunds, miniature Poodles, and Yorkshire Terriers [2, 3]. Slow progressive myxomatous changes in the mitral valve are the characteristic of this disease. The free edges of the leaflets become thickened and irregular, with areas showing bulging towards the left atrium (LA) [4].

Degeneration of the mitral valve is followed by mitral regurgitation and left-sided cardiac dilation. This cardiac remodeling eventually affects the mechanical functions of the heart, thereby leading to pulmonary congestion and reduced cardiac output. In humans, similar changes in the mitral valve and cardiac structure have been observed in diseased conditions. MMVD is an important cause of morbidity and mortality in humans and dogs [5]. MMVD is also known as degenerative mitral valve disease. As the word “degenerative” suggests, this disease is closely related to aging [6]. Therefore, appropriate management through early detection to slow the disease progression is important for increasing the lifespan. Currently, biomarkers such as N-terminal pro-brain natriuretic peptide (NT-pro BNP) and cardiac troponin I (cTnI) are being used as useful diagnostic tools.

Cartilage intermediate layer protein 1 (CILP1) is a non-collagenous protein present in the ECM and abundant in the articular cartilage [7]. Traditionally, CILP1 is considered to be associated only with cartilage-associated diseases. Recently, CILP1 has been detected in human cardiac tissue [8]. In rats, myocardial CILP1 levels are increased following aortic stenosis [9]. Furthermore, serum CILP1 levels are increased in diseases that induce myocardial remodeling in humans [10]. CILP1 acts as a TGF-β antagonist and interferes with the small mother against decapentaplegic (SMAD) signaling [11]. TGF-β is a pro-synthetic cytokine that stimulates extracellular matrix (ECM) production and regulates cell proliferation [12]. Overexpression of TGF-β isoforms in canines with MMVD has been reported in several studies [13, 14].

Herein, we evaluated serum CILP1 levels and correlated them with the disease stage and cardiac remodeling in dogs with MMVD. We also compared the diagnostic capability of CILP1 with that of conventional imaging and the biomarker NT-pro BNP. This study is the first to show the clinical application of CILP1 in veterinary medicine.

## Results

### Serum CILP1 levels in healthy controls and dogs with MMVD

**Fig. 1:**
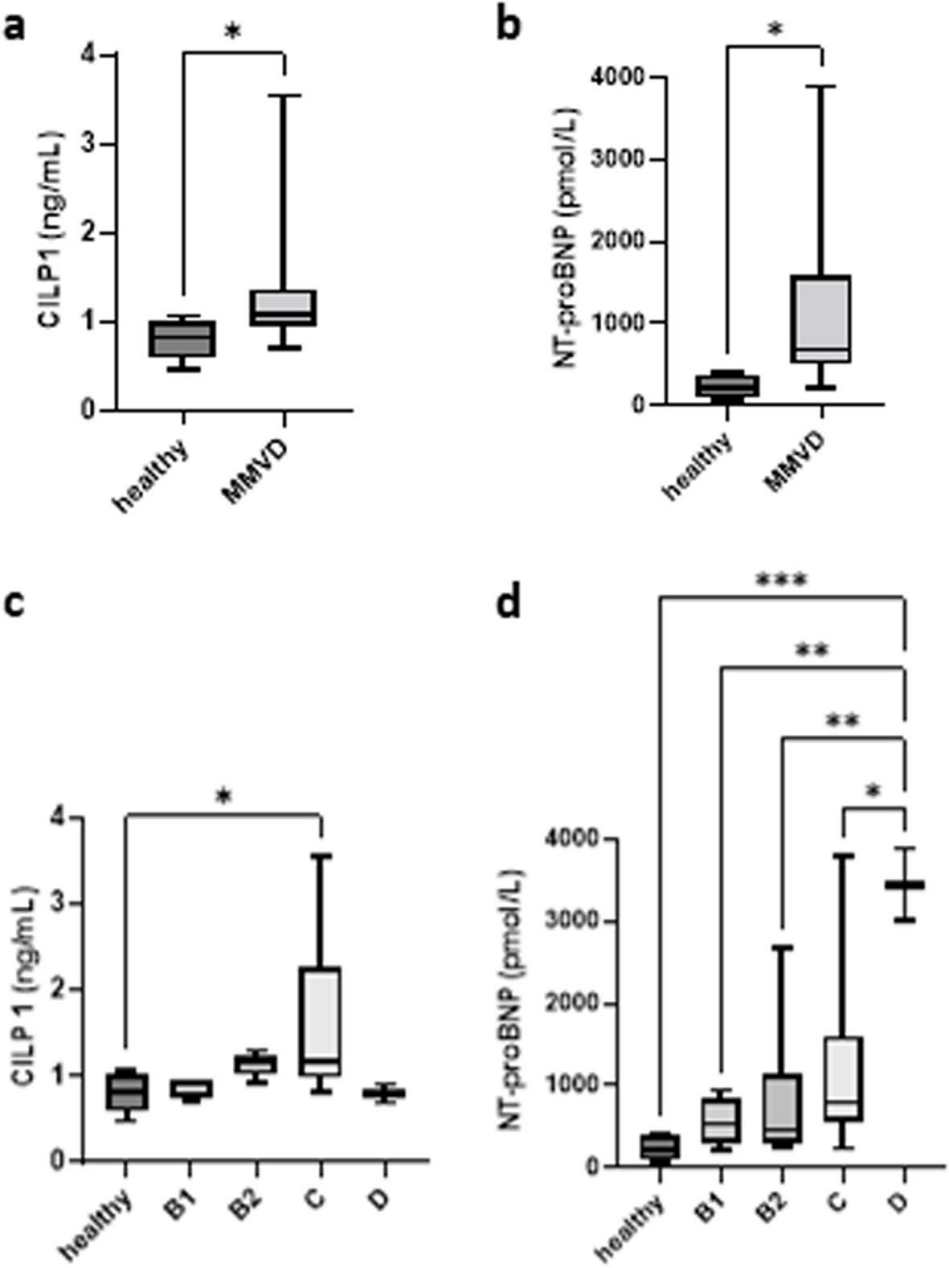
Boxplot of serum CILP1 and NT-proBNP levels in healthy controls and dogs with MMVD (**a**, **b**). Boxplot of serum CILP1 and NT-proBNP levels in healthy controls (*n* = 8) and dogs with MMVD staged according to the ACVIM consensus (**c**, **d**). Stage B1 (*n* = 4), stage B2 (*n* = 6), stage C (*n* = 15), and stage D (*n* = 2). The boxplot shows the average value along with the minimum and maximum values. ^*^P < 0.05, ^**^P < 0.01, and ^***^P < 0.001

Serum CILP1 levels were significantly higher in dogs with MMVD (1.352 ± 0.7662 ng/mL) compared to healthy controls (0.8046 ± 0.2379 ng/mL; *P* = 0.028) (Fig. 1a). NT-proBNP levels were significantly lower in healthy dogs (232.6 ± 134.8 pm/mol) compared to all those with MMVD (1202 ± 1127.9 pm/mol; *P* = 0.008) (Fig. 1b).

Serum CILP1 levels increased with the disease stage except for stage D. The mean concentration of serum CILP1 was 0.876 ± 0.122 ng/mL in stage B1, 1.136 ± 0.136 ng/mL in stage B2, 1.639 ± 0.928 ng/mL in stage C, and 0.796 ± 0.147 ng/mL in stage D. CILP1 level was significantly higher in stage C compared to the healthy control (*P* = 0.026) (Fig. 1c).

Serum NT-proBNP levels also increased with the disease stage. Stage C and stage D groups showed significantly increased levels compared to healthy controls. The mean concentration of serum NT-proBNP was 554.2 ± 295.8 pm/mol in stage B1, 798.1 ± 925.9 pm/mol in stage B2, 1238.3 ± 1050.2 pm/mol in stage C, and 3440 ± 626.1 pm/mol in stage D (Fig. 1d).

### Determination of sensitivity and specificity of CILP1 in MMVD patients

**Fig. 2:**
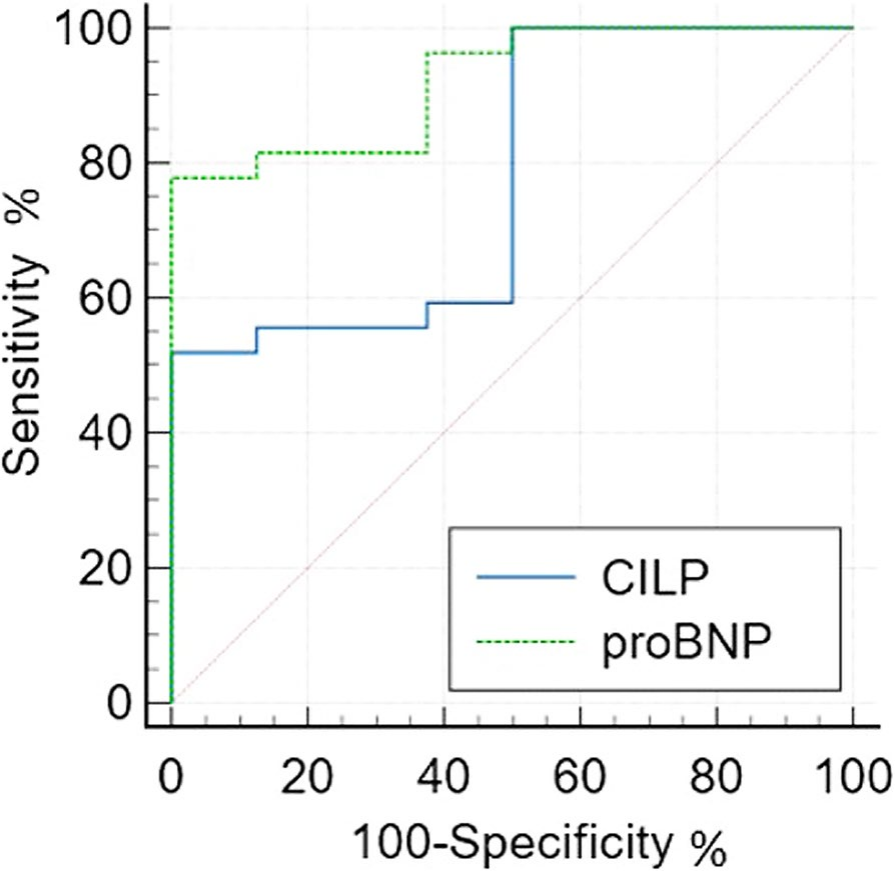
Comparison of receiver operating characteristic (ROC) plots of N-terminal pro-brain natriuretic peptide (NT-proBNP) and cartilage intermediate layer protein 1 (CILP1) for detecting MMVD in dogs

ROC curve analysis in dogs with MMVD and healthy controls showed that both CILP1 and NT-proBNP are good predictors of MMVD (CILP1; *P* = 0.004, NT-proBNP; *P* < 0.001). However, there was no significant similarity between the two ROC curves (*P* = 0.154) (Fig. 2).

Next, we evaluated the sensitivity and specificity of serum CILP1 levels for MMVD diagnosis. The cut-off value of CILP1 for detecting MMVD was found to be > 1.068 ng/mL (sensitivity 51.9%, specificity 100%). The area under the ROC curve (AUC) of CILP1 was found to be 0.921 (95%, CI: 0.762–0.984, *P* = 0.001).

### Relationship of CILP1 with vertebral left atrial score (VLAS), left atrial to aorta dimension (LA/Ao), and vertebral heart size (VHS)

**Fig. 3:**
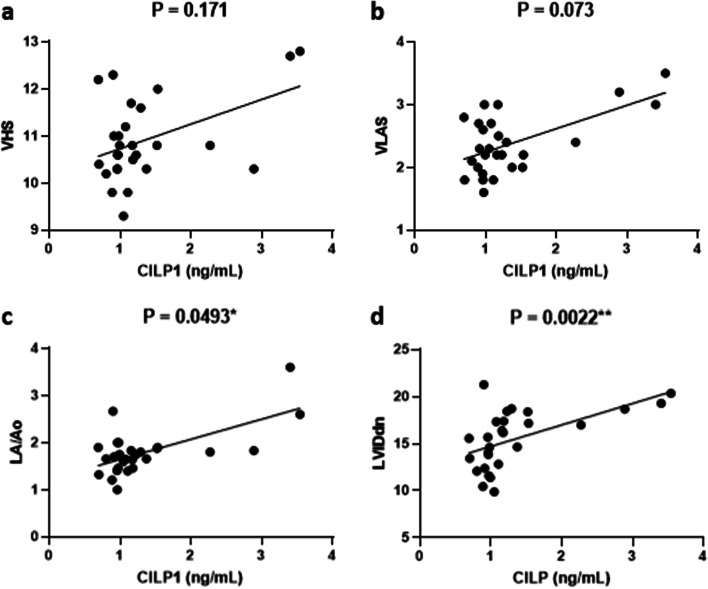
Correlation of CILP1 levels with VHS (**a**), VLAS (**b**), LA/Ao (**c**), and LVIDdn (**d**) in healthy controls and dogs with MMVD. Solid line represents a power trendline of the data. ^*^P < 0.05, ^**^P < 0.01, and ^***^P < 0.001

VHS, VLAS, LA/Ao, and LVIDdn were plotted for healthy controls and canines with MMVD using the scatter plot (Fig. 3). LVIDdn showed strongest relationship with CILP1 (*r* = 0.5628, *P* = 0.0022; Fig. 3d) followed by LA/Ao (*r* = 0.3820, *P* = 0.049). VHS (*r* = 0.2715, *P* = 0.171; Fig. 3a) and VLAS (*r* = 0.3505, *P* = 0.073; Fig. 3b) showed no association with CILP1.

### Comparison of diagnostic values of dogs with MMVD according to CILP1 cut-off value

**Fig. 4:**
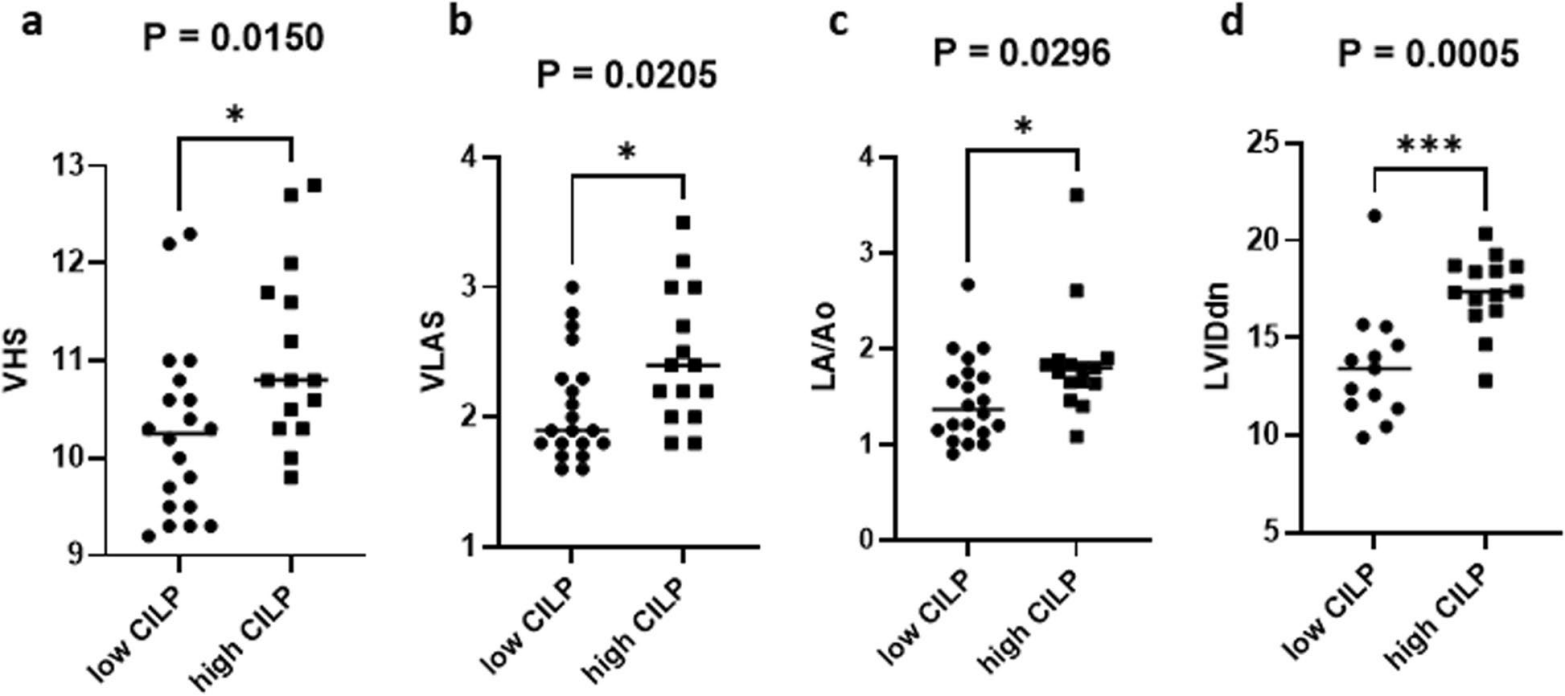
Box and scatter plots comparing VHS, VLAS, LA/Ao, and LVIDdn according to the CILP1 cut-off values in the MMVD group. Horizontal bars represent the mean value. ^*^P < 0.05, ^**^P < 0.01, and ^***^P < 0.001

The cardiac diagnostic values were compared according to CILP1 cut-off value in whole cohort (Table 1). Dogs with high serum CILP1 levels (> 1.068 ng/mL) showed increased VHS, VLAS, LA/Ao, and LVIDdn than those with low serum CILP1 levels (< 1.068 ng/mL). VHS (*P* = 0.0150; Fig. 4a), VLAS (*P* = 0.021; Fig. 4b), and LA/Ao (*P* = 0.030; Fig. 4c) showed a significant correlation with CILP1 levels (*P* = 0.022). Moreover, LVIDdn showed the strongest association with CILP1 levels (*P* = 0.001; Fig. 4d).

**Table 1:** The diagnostic performance of CILP1 according to the cut-off value

	CILP1 < 1.068 ng/mL	CILP1 > 1.068 ng/mL	*P*-value
Dogs (N)	13	14	
Age (years)	9.75 ± 1.70	11.75 ± 2.52	0.6778
Heart rate (bpm)	124.00 ± 12.56	145 ± 28.84	0.4298
Blood pressure (mmHg)	130.00 ± 14.14	142.37 ± 22.01	0.3304
Murmur grade	4 ± 0.81	4.33 ± 0.70	0.0671
Radiology			
VHS	10.72 ± 0.66	10.88 ± 0.85	0.0150^*^
Vlas	2.45 ± 0.59	2.36 ± 0.43	0.0205^*^
Echocardiology			
LVFS	59.52 ± 4.52	54.80 ± 9.93	0.7287
LVIDdn	22.82 ± 2.14	22.43 ± 3.50	0.0005^**^
LVIDsn	9.25 ± 0.81	10.31 ± 2.53	0.1037
LA/Ao	1.66 ± 0.45	1.79 ± 0.49	0.0296^*^
E-wave	92.70 ± 17.42	84.70 ± 23.67	0.8238
E/A	0.98 ± 0.22	0.95 ± 0.26	0.3137

*CILP1* Cartilage intermediate layer protein 1, *VHS* Vertebral heart size, *Vlas* Vertebral left atrial score, *LVFS* Left ventricular fraction shortening, *LVIDdn* Left ventricular end diastolic diameter normalized for body weight, *LVIDsn* Left ventricular end systolic diameter normalized for body weight, *LA/Ao* Left atrial to aortic root ratio, *E* Early diastolic inflow velocity, *A* late diastolic inflow velocity, *NT-proBNP* N-terminal pro-B-type natriuretic peptide (NT-proBNP)

^*^P < 0.05

**P < 0.001

## Discussion

Recently, CILP1 has gained importance as a biomarker of cardiac diseases in humans and mice. Several studies have evaluated CILP1 as a marker of cardiac remodeling [9, 10]. To the best of our knowledge, this study is the first to show the importance of CILP1 in veterinary medicine. Our study is meaningful because canines were carefully classified based on MMVD staging, and a specific comparison between cardiac remodeling parameters and biomarkers was performed.

CILP1 inhibits Smad3 activation and myofibroblasts differentiation and suppresses the expression of TGF-β [9]. CILP1 is present in the ECM and interacts with TGF-β via the feedback mechanism which is associated with cardiac fibrosis [10]. By inhibiting the TGF-β signaling activity, CILP1 slows down ECM accumulation and prevents further development of cardiac fibrosis [11]. These findings suggest that CILP1 can be a potential marker of TGF-β-induced cardiac fibrosis. We observed that CILP1 levels are significantly increased in stage C of MMVD, but not in stage D. We speculate that increased CILP1 levels would have been observed in stage D MMVD if the sample size was bigger.

Furthermore, it would have been more appropriate if echocardiographic structural parameters were used rather than American College of Veterinary Internal Medicine (ACVIM) classification. Moreover, increased cardiac remodeling based on structure parameters and higher CILP1 levels were observed in our study suggesting that CILP1 could be a biomarker of cardiac remodeling in canine MMVD.

LA volume has strong predictive power for MMVD in dogs [15]. We observed a correlation of CILP1 levels with LVIDdn and LA/Ao. Furthermore, we confirmed the association of CILP1 only with echocardiographic values and not with radiological values. Although VHS and VLAS showed no significant relationship with CILP1, both of them showed significance when MMVD was classified according to the cut-off value. This suggests that if appropriate CILP1 cut-offs are used, this protein could act as a biomarker of the left heart remodeling in MMVD.

The CILP1 pathway could be involved in preventing excessive fibrosis by inhibiting the profibrotic TGF-β signaling in the myocardium [10]. Moreover, the binding of CILP1 to TGF-β may influence the circulating CILP1 levels, which might be the reason for underestimated CILP1 levels reported in a previous study [16]. This could explain the fact that although a weaker upregulation of CILP1 expression was observed in a particular stage of the disease, LV fibrosis was more pronounced.

A previous study suggests that in mice, CILP1 expression is more associated with RV pressure overload than LV pressure overload since a 26-fold up-regulation of CILP1 was observed following RV pulmonary artery constriction compared to a fivefold up-regulation following transverse aortic constriction [17]. Further studies are needed to confirm the correlation between the remodeling of specific cardiac structures in canine MMVD and CILP1.

Two of the most widely used cardiac biomarkers in canines are cTnI and NT-proBNP [18, 19]. cTnI is released extracellularly from damaged cardiomyocytes and affects heart contraction and relaxation [20]. NT-proBNP release is due to mechanical stress and stretching of the cardiac muscles [21]. However, cTnI and NT-proBNP levels are also increased in non-cardiac diseases such as kidney disease, pancreatitis, pyometra, viral or parasitic infection, and pulmonary hypertension [20, 22, 23]. Thus, a combination of biomarkers is more effective than single markers for risk prediction of heart disease in humans [24]. In veterinary medicine, NT-proBNP and cTnI can independently predict survival and can provide important information on prognosis. Therefore, to predict survival time, measuring both NT-proBNP and cTnI together will be more helpful than measuring NT-proBNP alone [25]. Therefore, there is always a need for identifying additional biomarkers.

Our study has several limitations. Breeds with MMVD overlap with chondroscopic breeds. Although dogs with cartilage-associated diseases were excluded from this study, it is well established that CILP1 is a cartilage-related protein, which could be a limitation of this study. Another limitation of our study is the small sample size in each group, especially 2 cases in stage D group cannot make any statistics. Thus, further studies with a larger sample size are required to investigate the prognostic value of CILP1 and its correlation with cardiac remodeling. Moreover, we compared the MMVD group with the healthy control group only. However, other non-cardiac diseases are also associated with CILP1, including osteoarthritis, intervertebral disc degeneration, and diabetes mellitus [26–28]. Although none of these diseases are associated with increased circulating CILP1 levels, additional studies are needed to determine whether CILP1 can be used to differentiate between cardiac and non-cardiac diseases.

## Conclusion

CILP1 is useful for detecting cardiac remodeling caused by MMVD. Furthermore, CILP1 might be a biomarker of ACVIM stages A, B, and C in canine MMVD. Further studies with a larger sample size are required to investigate the usefulness of CILP1 levels for stage D.

## Methods

### Case selection and medical records review

Twenty-seven client-owned dogs diagnosed with MMVD at the veterinary teaching hospital were included in the disease group. Four Beagles and four client-owned dogs above seven years of age with a good health history and no abnormal findings were included as healthy controls. The study protocol was approved by the Institutional Animal Care and Use Committee of Chonnam National University (CNU IACUC-YB-2019–23). Owners of all included dogs provided written informed consent.

A complete physical and radiological examination along with echocardiology was performed on all dogs. Dogs with heart diseases other than MMVD, such as congenital heart disorder, myocardial disease, and right heart failure were excluded [29].

### Study population

Dogs were staged according to the ACVIM consensus guidelines for the diagnosis of MMVD (Table 2) [29]. Among all MMVD dogs, 11% (4/30) were in stage B1, 17% (6/30) in stage B2, 43% (15/30) in stage C, and 6% (2/30) were in stage D MMVD (Table 3).

**Table 2:** Staging system for MMVD according to the ACVIM MMVD consensus

Stages	Explanation
A	Dogs at a high risk but currently have no disorder
B1	Asymptomatic dogs having no cardiac remodeling
B2	Asymptomatic dogs with left atrial and ventricular enlargement
C	Dogs with clinical signs of heart failure due to MMVD
D	Dogs with end-stages MMVD, in which clinical sings of heart failure are refractory to the standard treatment

**Table 3:** Number of dogs in different groups

Group		N	%
Healthy control		8	23
MMVD		27	77
MMVD stages	B1	4	11
	B2	6	17
	C	15	43
	D	2	6

### Blood sampling and processing

For evaluating serum NT-proBNP and CILP1 levels, 4 mL of the blood was collected from the jugular vein of dogs into a gel separator tube. The serum was separated by centrifugation at 3000 × g for 10 min within 2 h after collection. Samples were stored at -80 °C or in dry ice during transport to the laboratory for measurements. Serum NT-proBNP levels were determined using the VET Chroma Analyzer (Anivet diagnostics Inc, South Korea). Serum CILP1 levels were measured using a commercially available ELISA kit (Canine Cartilage intermediate layer protein 1 Elisa Kit, MyBioSource.com, California, USA). Analysis was performed according to the manufacturer's instructions.

### Cardiac evaluation through thoracic radiography and echocardiography

The cardiac size was evaluated by measuring the VHS and VLAS on thoracic radiography in all dogs. Transposing the heart's long and short axes onto the vertebral column, the number of vertebrae starting at the cranial border of T4 was recorded. The VHS was then calculated by adding these values [30]. The first line was drawn from the center of the most ventral aspect of the carina (bifurcation of the left and right mainstem bronchi) to the most caudal aspect of the left atrium where it intersects with the dorsal border of the caudal vena cava. Starting at the cranial border of T4 and extending caudally just ventral and parallel to the vertebral canal, the second line of identical length to the first was drawn. The VLAS was then calculated as the length of the second line and expressed in terms of vertebral-body units to the nearest 0.1 vertebrae [31].

Echocardiography measured the following six variables: left ventricular fraction shortening (LVFS), left ventricular-end diastolic diameter normalized for the body weight (LVIDdn), left ventricular end-systolic diameter normalized for body weight (LVIDsn) measured by the M-mode on the right parasternal short-axis view [32], left atrial to aortic root ratio (LA/Ao) measured on the right parasternal short-axis view [33], early diastolic inflow velocity (E wave), early diastolic inflow velocity to late diastolic inflow velocity ratio (E/A) measured by pulsed-wave Doppler on the left apical 4-chamber view [34]. The examination was performed by a single operator using the General Electric Logiq V5 EXPERT machine (GE Medical Systems, Wuxi, China).

### Statistical analysis

Statistical analyses were performed using GraphPad Prism version 9 (GraphPad Software, La Jolla, CA, USA) and MedCalc Statistical Software (version 20.011; MedCalc Software bvba, Ostend, Belgium). The data were tested for normal distribution using the Shapiro–Wilk test. The comparison of CILP1 levels between different MMVD stage groups was performed using the Kruskal–Wallis test. Dunn’s multiple comparison test was used to determine statistically significant differences between groups. An unpaired t-test was used to compare cardiac remodeling values and cardiac biomarkers between the MMVD group and healthy dogs. A *P-*value less than 0.05 was considered statistically significant. The simple linear regression analysis was used to evaluate the relationship between CILP1 and other diagnostic parameters, including cardiac biomarkers and cardiac remodeling values (echocardiographic and radiographic parameters). The significance was determined by the coefficient and a *P-*value < 0.05. Receiver operating characteristic (ROC) curve analysis was performed to evaluate the predictive value of CILP1 according to the MMVD stage. The optimal CILP1 cut-off concentration for predicting MMVD was determined using the Youden index.

## Data Availability

Additional data can be provided by the corresponding author upon reasonable request.

## Abbreviations

MMVD: Myxomatous mitral valve degeneration
CILP1: Cartilage intermediate layer protein 1
ROC: Receiver operating characteristic
LVIDdn: Left ventricular end diastolic diameter normalized for body weight
LA/Ao: Left atrial to aorta dimension
VHS: Vertebral heart size
VLAS: Vertebral left atrial score
LA: The left atrium
NT-pro BNP: N-terminal pro-Brain Natriuretic Peptide
cTnI: Cardiac troponin I
SMAD: Small mother against decapentaplegic
ECM: Extracellular matrix
AUC: The area under the ROC curve
ACVIM: American College of Veterinary Internal Medicine
NRF: The National Research Foundation of Korea
LVFS: Left ventricular fraction shortening
LVIDsn: Left ventricular end-systolic diameter normalized for body weight
